# The impact of offspring and maternal obesogenic diets on adult offspring oocyte mitochondrial morphology in primordial and preantral follicles

**DOI:** 10.1371/journal.pone.0305912

**Published:** 2024-06-27

**Authors:** Inne Xhonneux, Waleed F. A. Marei, Ben Meulders, Jens Slootmans, Isabel Pintelon, Jo L. M. R. Leroy

**Affiliations:** 1 Department of Veterinary Sciences, Laboratory of Veterinary Physiology and Biochemistry, Gamete Research Centre, University of Antwerp, Wilrijk, Belgium; 2 Faculty of Veterinary Medicine, Department of Theriogenology, Cairo University, Giza, Egypt; 3 Department of Biosystems, University of Louvain, Louvain, Belgium; 4 Flanders Research Institute for Agriculture, Fisheries and Food, Merelbeke, Belgium; 5 Department of Veterinary Sciences, Laboratory of Cell Biology and Histology, University of Antwerp, Wilrijk, Belgium; 6 Antwerp Centre for Advanced Microscopy (ACAM), University of Antwerp, Wilrijk, Belgium; University of Hawai’i at Manoa, UNITED STATES

## Abstract

Diet-induced obesity reduces oocyte quality mainly by impacting oocyte mitochondrial functions. Moreover, maternal obesity is associated with mitochondrial dysfunction in oocytes of their adult offspring. However, these effects were reported only in fully grown oocytes, mainly in the form of abnormal mitochondrial ultrastructure. It is unknown if obesogenic (OB) diets or maternal obesity already impact the primordial and preantral follicles. Considering the long duration and dynamics of folliculogenesis, determining the stage at which oocytes are affected and the extent of the damage is crucial for optimal reproductive management of obese patients and their daughters. Potential interaction between maternal and offspring diet effects are also not described, yet pivotal in our contemporary society. Therefore, here we examined the impact of OB diets on oocyte mitochondrial ultrastructure in primordial and activated preantral follicles in offspring from diet-induced obese or lean mothers. We used an outbred Swiss mouse model to increase the pathophysiological relevance to humans. Female mice were fed control or OB diets for 7 weeks, then mated with control males. Their female offspring were fed control or OB diets after weaning for 7 weeks (2-by-2 factorial design). Adult offspring ovarian sections were examined using transmission electron microscopy. We characterised and classified unique features of oocyte mitochondrial ultrastructure in the preantral follicles. An increase in mitochondrial matrix density was the most predominant change during follicle activation in secondary follicles, a feature that is linked with a higher mitochondrial activity. Maternal obesity increased mitochondrial density already in the primordial follicles suggesting an earlier increase in bioenergetic capacity. Maternal obesity did not induce abberant ultrastructure (abnormalities and defects) in primordial or preantral follicles. In contrast, offspring OB diet increased mitochondrial abnormalities in the primordial follicles. Further investigation of the consequences of these changes on oocyte metabolic regulation and stress levels during folliculogenesis is needed.

## Introduction

The growing global prevalence of obesity has raised significant concerns regarding its detrimental consequences on health and fertility, and its effects across generations. Obese women have an increased risk of subfertility, involving endocrine imbalance, ovulatory malfunction, polycystic ovarian disorders, and reduced oocyte quality [1,2]. Moreover, children born to obese mothers have increased risk of developing metabolic syndrome, including obesity and diabetes [3], and are thus more prone to the development of metabolic diseases later in life [4–7]. A few studies in diet-induced obese mouse models suggested that maternal obesity increases the risk of reproductive disorders in the offspring as well [8–10]. Since children mostly follow the same lifestyle and dietary habits of their parents [11], some of the reported effects of maternal obesity on offspring health and fertility can be, at least in part, due to direct effects of consuming obesogenic (OB) diets after ablactation. Dissecting these effects and investigating the potential interaction between offspring’s OB diet and the maternal metabolic background became crucial in our contemporary society, in which most obese woman are born to obese mothers [12–14].

Reduced oocyte quality in diet-induced obesity has been described in several studies and is linked with low maturation and fertilization rates, reduced embryo developmental competence and low pregnancy success rates after ART treatments [15,16]. This was usually attributed to biochemical alterations in the follicular fluid after antrum formation [17–19]. We and others have shown that mitochondrial dysfunction plays a key role in the pathogenesis of reduced oocyte quality. Consumption of OB diets was shown to alter the oocyte mitochondrial ultrastructural morphology, for example by increasing mitochondrial vacuolization and altering inner membrane organization, or by inducing mitochondrial elongation [20–22]. This was associated with concurrent alterations in mitochondrial inner membrane potential, reduced ATP production and increased mitochondrial reactive oxygen species (ROS) production [23–25]. Importantly, most of these studies examined the effect of obesity on oocyte quality in fully grown oocytes either in the preovulatory follicles or after ovulation [26]. However, it is not known if the dormant primordial pool and early preantral follicular stages are also affected. Folliculogenesis is a lengthy process which involves highly complex cytoplasmic changes in organelle structure and functions, including mitochondrial replication and gradual increase in mitochondrial bioenergetic activities [27]. This is crucial to meet the increasing metabolic demand to support cytoplasmic and nuclear maturation in the oocyte and grant oocyte developmental competence [28]. Determining the stage at which oocytes are affected is crucial to optimize reproductive management of obese patients by e.g. optimizing the duration or timing of the interventions during the preconception period.

There are several indications that suggest that preantral follicles are vulnerable to metabolic stress. This is most clearly described in the dairy cow model. High yielding dairy cows suffering from severe negative energy balance during the early post-partum period and during heat stress show elevated concentrations of lipotoxic fatty acids and reactive oxygen species in the blood and follicular fluid, which induces oocyte mitochondrial dysfunction and reduced oocyte quality, similar to those described in obese women [29–31]. Nevertheless, it was noticed that the reduced oocyte quality persists for a few months after the restoration of energy balance and alleviation of stress [32]. According to the Britt’s hypothesis [33], this suggests that early preantral stages of follicular development are also affected. Recent data could provide strong evidence that is in line with this notion [34]. Similarly, studies from our laboratory recently indicated that an increased rate of mitochondrial ultrastructural abnormalities is still detectable in diet-induced obese mice after 4–6 weeks of diet normalization or caloric restriction, implying that the dormant follicle pool may indeed be affected [35]. Nevertheless, the nature and extent by which oocyte mitochondria can be affected by obesogenic diets in primordial and preantral follicles has never been investigated.

In addition, maternal obesity is also associated with mitochondrial dysfunction in oocytes of their offspring, mainly in the form of abnormal mitochondrial ultrastructure [36]. Again, this was only described in fully grown oocytes from preovulatory follicles in the adult offspring, while the occurrence of these defects in the primordial pool was not examined. This fundamental knowledge, and the potential interaction with the direct effects of the offspring’s diet can be useful to develop more efficient strategies to prevent and treat subfertility in females born to obese mothers.

Therefore, in this study we hypothesized that both maternal and offspring OB diet consumption can affect the oocyte mitochondrial morphology in the primordial and early preantral ovarian follicles in the offspring. We also hypothesized that the effects of the offspring diet may depend on the maternal dietary background. To test these hypotheses, we used a diet-induced obese mouse model in which mothers and their female offspring were fed a control or high-fat high sugar diet in a 2-by-2 factorial design. This design enabled us to address the interaction between the effect of the maternal and offspring OB diet, mimicking the same pattern of female familial obesity in our society. Outbred Swiss mice were used to increase the pathophysiological relevance to the human physiology, as validated and described in our previous studies [37,38]. While examining mitochondrial parameters in oocytes of preantral follicles can be challenging, we used transmission electron microscopy to investigate mitochondrial morphology, which also enables precise identification of the corresponding stage of follicular development. Oocytes of the primordial and early activated follicles (primary and secondary follicles) were examined in ovarian sections of adult offspring. Since studies describing mitochondrial morphology in early developing follicles are lacking, it was necessary to first provide a comprehensive characterization and classification of the normal mitochondrial ultrastructure in oocytes of primordial and early activated follicles of the control adult offspring.

## Materials & methods

### Experimental design

**Fig 1:**
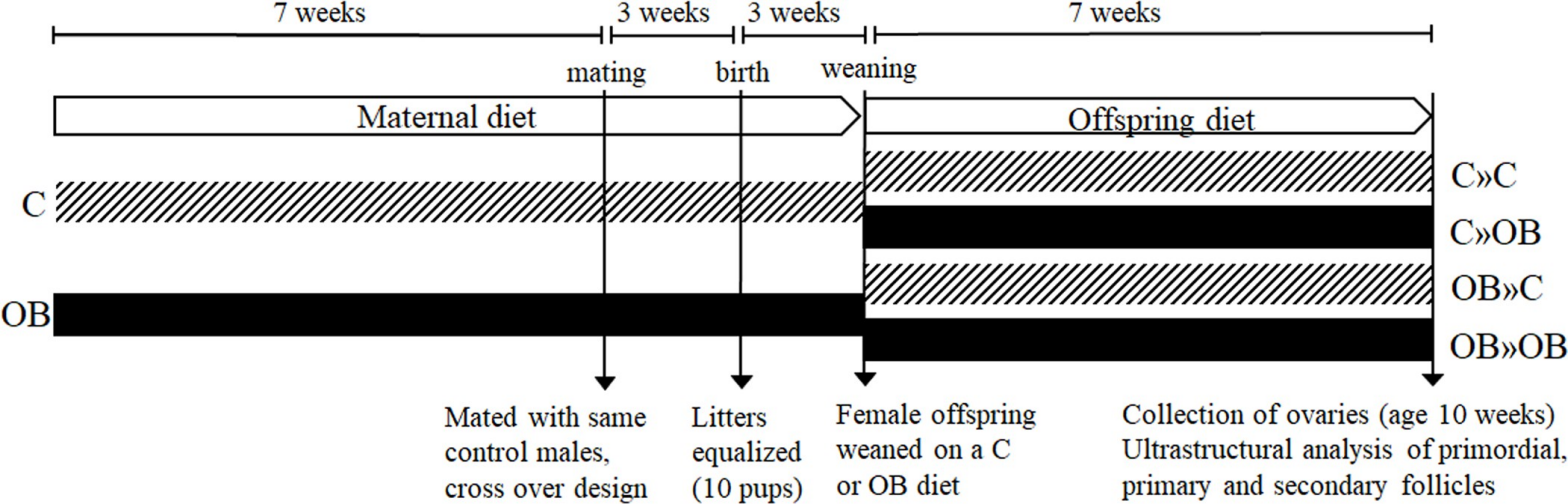
Schematic 2-by-2 factorial design of the study. Offspring were fed a control (C) or an obesogenic diet (OB) and born to mothers that were either fed a C or OB diet, in a 2-by-2 factorial design. Offspring treatment groups are named as MaternalDiet»OffspringDiet.

This study was approved by the Ethical Committee for Animal Testing and performed in accordance with the ARRIVE guidelines. The protocol was approved by the Committee on the Ethics of Animal Experiments of the University of Antwerp (Protocol Number: ECD 2018–05). All efforts were made to minimize animal suffering. Female Swiss mice (age 3 weeks) were fed a C diet (10% fat, 7% sugar, Sniff diets D12450J, containing 10% fat and 7% sugar (E157453-04), n = 6) or an OB diet (Sniff diets E15741-34, 60% fat (beef Tallow), 20% sugar, n = 6) for 7 weeks, then mated with the same Swiss males (n = 2) fed a standard chow diet in a cross-over design. Their weight gain was recorded weekly during the trial. All mice stayed on their corresponding diet during pregnancy and lactation. Litter sizes were recorded and equalized to 10 pups to avoid unequal nutrient supply between different litters [39,40]. Female offspring were randomly and equally weaned onto either a C or an OB diet at 3 weeks of age, creating a 2-by-2 factorial design and resulting in four treatment groups named as MaternalDiet»OffspringDiet: 1) C»C: C-born C-fed pups 2) C»OB: C-born OB-fed pups 3) OB»C: OB-born C-fed pups and 4) OB»OB: OB-born OB-fed pups (Fig 1). They were fed their corresponding diet for 7 weeks after weaning and were weighed weekly. At 10 weeks, the oestrus cycles of the mice were synchronized using the Whitten-effect 12 h before euthanasia and sample collection [41]. Each pup was euthanized by cervical dislocation. One ovary per offspring per mother (n = 6 per group) was collected and immediately fixed in sodium cacodylate-buffered glutaraldehyde solution. Random ultrathin ovarian sections were examined for mitochondrial ultrastructure using TEM. All primordial, primary, and secondary follicles in each section were examined. To compare the oocyte mitochondrial morphology in preantral follicles with the already well described mitochondrial morphology of mature (ovulated) oocytes, few ovulated oocytes were collected of adult C»C offspring, according to the protocol described by Xhonneux [25].

### Sample collection and preparation

Immediately post-collection, ovaries were fixed in 0.1 M sodium cacodylate-buffered (pH 7.4) 2.5% glutaraldehyde solution with 0.05% CaCl_2_.2H_2_0 at 4°C. The fixative was removed, and the tissue was rinsed in 0.1 M sodium cacodylate-buffered (pH 7.4) 0.05% CaCl_2_.2H_2_O, 7.5% sucrose solution (RT). Post-fixation tissue was incubated (2 h, RT) in 1% osmium tetroxide (OsO_4_) in a 0.033 M veronal acetate buffer containing 4% sucrose. The tissue was rinsed with 0.05 M veronal acetate buffer (pH 7.4) containing 6% sucrose RT after OsO_4_ removal. Afterwards, 1% tannic acid staining was performed in veronal acetate 6% sucrose (1 h, RT) and the samples were washed thoroughly with veronal acetate 6% sucrose, followed by dehydration using an ethanol gradient starting from 50% to 100% ethanol. Dehydration was continued with propylene oxide for 30 minutes. To impregnate the samples, the tissue was treated overnight with a propylene oxide/EMBed 812 resin (EMBed-812 Kit, E14120) mixture (1:3) without accelerator (DMP-30), while on a rotary shaker (RT). Subsequently, the sample was treated with EMBed 812 resin mixture (without accelerator) for 2 x 2 h at 37°C, followed by EMBed 812 resin mixture with accelerator (1 h, 37°C). Before the sample was embedded, it was transferred into a gelatine capsule or into a flat embedding mold, then covered with EMBed 812 resin mixture containing accelerator to allow polymerization (36 h, 65°C). Afterwards, ultrathin sections (50 nm thick) were cut using Ultramicrotome (Leica EM UC7) with diamond knife (ultra 45°, Diatome), then stained with lead citrate lead citrate, rinsed with 0.05 M sodium hydroxide and CO_2_-free ultrapure water and examined with TEM Tecnai G2 Spirit Bio TWIN microscope (Fei, Europe BV, Zaventem, Belgium) at 120 kV. All primordial, primary, and secondary follicles present in one or two random section(s) midway through the ovary (with both ovarian medulla and cortex present) were assessed. Non-overlapping pictures were taken of all mitochondria present in the oocytes of these follicles. All mitochondria per oocyte section were morphologically evaluated and classified by two independent experienced assessors blind to the treatment groups.

### Classification of the follicles

**Fig 2:**
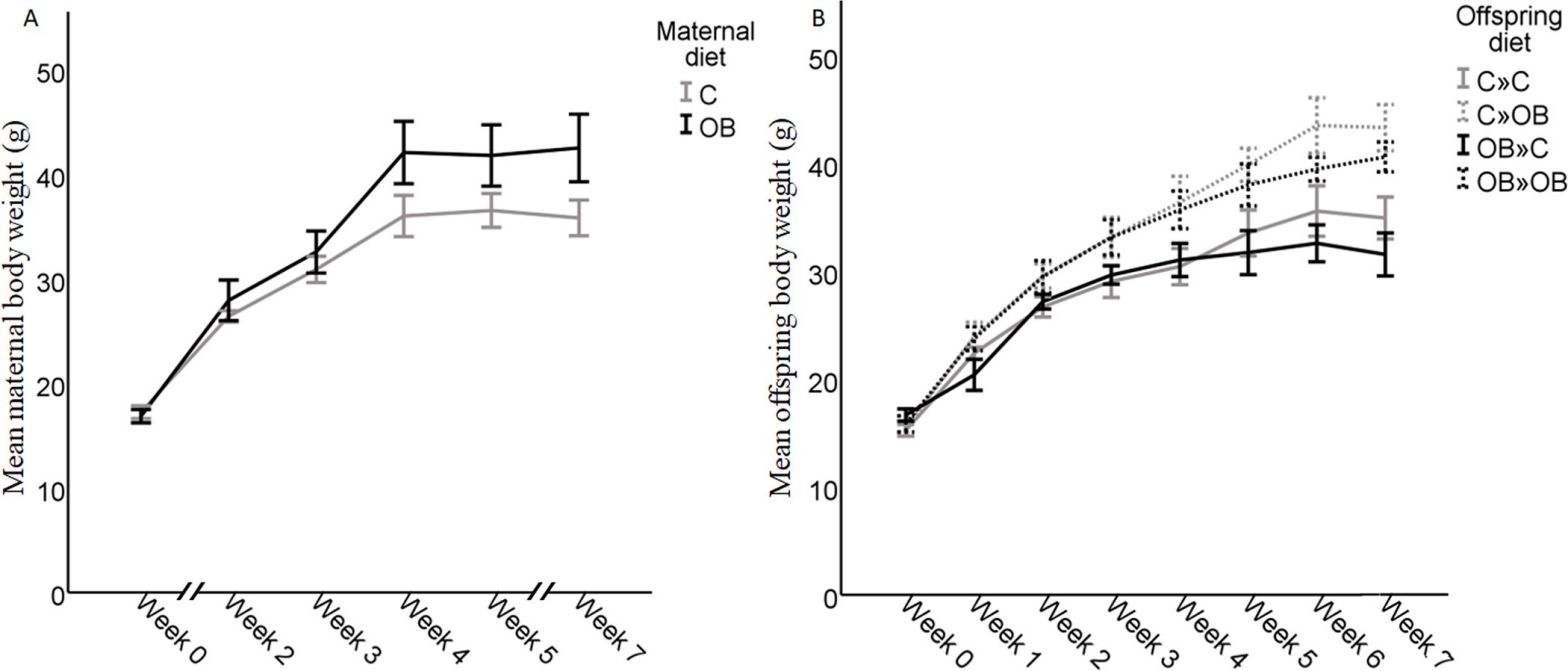
Transmission electron microscopic overview of assessed follicles (C»C) in this study. **A**. Primordial follicle **B**. Primary follicle **C**. Secondary follicle (O = oocyte; ON = oocyte nucleus; ZP = zona pellucida; GC = granulosa cells).

Follicles were classified as 1) Primordial, when the oocyte is surrounded by a single layer of flattened (pre-) granulosa cells, 2) Primary, surrounded by one layer of cuboidal granulosa cells, 3) Secondary, surrounded by two to four layers of granulosa cells without antrum [42] (Fig 2). Primordial follicles are also referred to as dormant follicles. After follicle activation occurs, the primary and secondary follicles are also referred to as activated or growing follicles. Follicles were excluded from the analysis when signs of follicular atresia were evident, such as oocyte fragmentation, granulosa cell pyknosis, degeneration, and damaged cell membrane, as described by Yu [43] and Regan [44] (see example of atretic follicles in S1 Fig).

### Classification of oocyte mitochondria within follicles

Since studies investigating mitochondrial morphology in the preantral follicles of adult mice are lacking, we have first characterized and classified different oocyte mitochondria according to their ultrastructure. This was done using the preantral follicle oocytes of the C»C pups as a reference. The mitochondrial morphology was classified based on matrix density (dense or light), shape (spherical or non-spherical), the presence of cristae (not present, sparse or abundant), and the presence of a vacuole. Differences in mitochondrial density, shape, or presence of cristae are not considered abnormalities at these preantral follicle developmental stages, and can rather imply acquisition of bioenergetic function [45–47]. In addition, other specific ultrastructural features which clearly illustrate an abnormality or defect in the oocyte mitochondrial were classified separately, such as mitochondria showing loose inner membranes, broken or protruded outer membranes, rose-petal shaped mitochondria, enlarged mitochondria (>1μm), and mitochondria having electron dense foci. These forms were collectively classified here as abnormal mitochondria, as described in mature oocytes by Marei [20] and Xhonneux [25].

### Statistical analysis

Statistical analysis was done using IBM SPSS Statistics 29 (for Windows, Chicago, IL, USA). Data were checked for equality of variance (Levene’s Test) and normality of distribution (residual QQ-plots).

Differences in weight of the mothers at mating (after 7 weeks of being fed their corresponding diet) were analyzed using Mann-Whitney-U, since the data were not homogenous in variance.

Effect of the maternal diet on litter sizes and differences in offspring weight at birth and at weaning were investigated using Independent Sample T-test.

Changes in mean live body weight (recorded weekly) of the mothers and their offspring were compared using Repeated Measures ANOVA.

The maternal and offspring diet effect and their interaction on offspring weight at adulthood (age 10 weeks) was tested using Two-way ANOVA. If the interaction was not significant, it was omitted from the model.

Differences in the proportions of different classes of mitochondrial ultrastructure between follicular stages (from primordial to secondary follicular stage) within the control offspring (C»C) were examined using generalised linear mixed models (binary logistic regression) with follicle stage as factor. Correction for nesting of the data collected from multiple follicles within the same offspring ovary was always implemented in the statistical analysis, blocking for differences in number of follicles per offspring.

To estimate the effect of the maternal and offspring diets and their interplay on the mitochondrial morphology (proportion of different mitochondrial ultrastructural classes) in oocytes of preantral follicles generalised linear mixed models (binary logistic regression, with diet as factor) was used. If the interaction was not significant, it was omitted from the model.

Differences with *P*-values ≤ 0.05 are reported as statistically significant, and 0.05 < *P*-values ≤ 0.1 are reported as tendencies. Numerical data are presented as mean ± S.E., while categorical data are presented as proportion percentage ± S.E.

An overview of the number of mitochondria assessed in each follicular stage in each group is presented in S1 Table 1–3 in S1 File. All proportions are summarized in S2 Table 1–3 in S1 File.

## Results and discussion

### Maternal and offspring body weight

**Fig 3:**
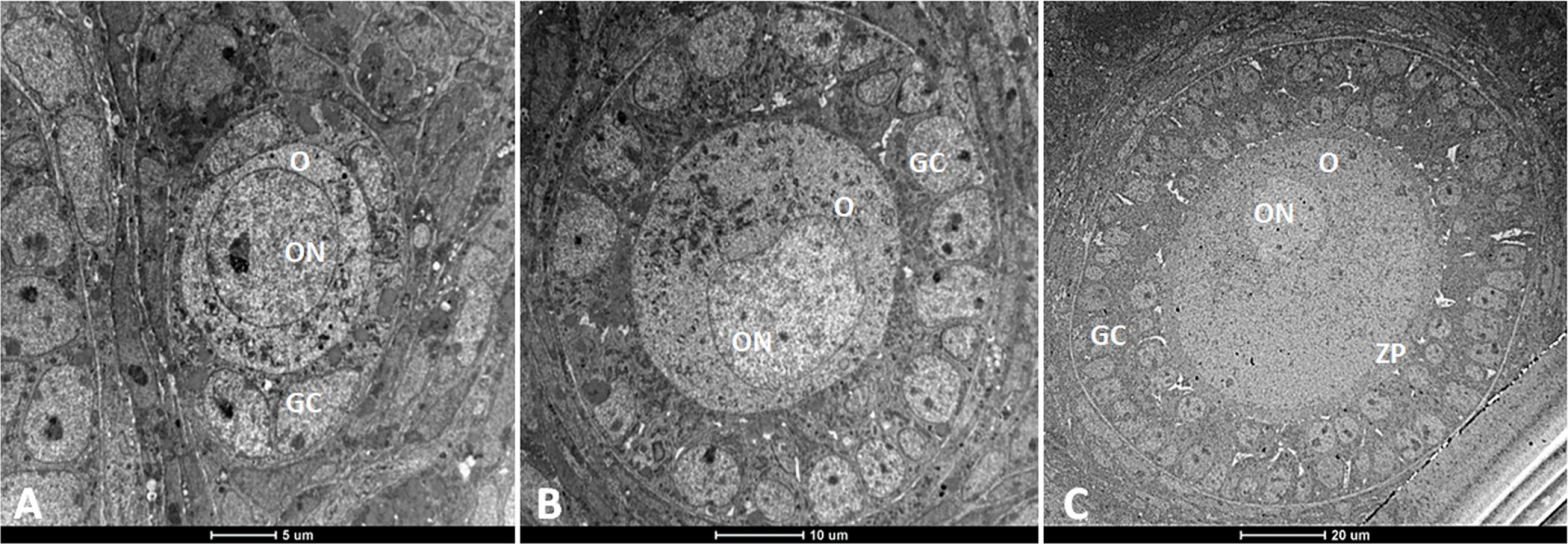
Growth curve of the mothers (A) fed a C or OB diet, and their offspring after weaning (B) in the different treatment groups where C or OB-born offspring were fed a C or an OB diet, in a 2-by-2 factorial design. Data are presented as mean±S.E.M. and are derived from 6 mothers fed a C or OB diet and one offspring per mother (n = 6 offspring/group).

The OB mothers weighed significantly more at mating (after 7 weeks on diet) compared to the controls (C: 35.89 ± 1.71 *vs*. OB: 42.53 ± 3.23, g, *P* = 0.020), but this did not impact the litter sizes (C: 15 ± 0.82 *vs*. OB: 13 ± 0.86, pups/litter, *P* = 0.122). Therefore, litter size equalization was similar in all groups. The maternal growth curve is shown in Fig 3A.

The maternal OB diet did not increase offspring weight at birth (C: 2.40 ± 0.01, OB: 2.37 ± 0.12, g, *P* = 0.650), at weaning (C: 15.36 ± 0.40; OB: 16.27 ± 0.46, g, *P* = 0.150), or at adulthood (at sample collection, after 7 weeks on diet, C»C: 35.60 ± 1.95 *vs*. C»OB: 43.34 ± 2.15, and OB»C 31.57 ± 1.99 *vs*. OB»OB 40.62 ± 1.38, g, *P* = 0.113).

Offspring weight at adulthood was increased by offspring OB diet (*P* < 0.001), which was not dependent on the maternal dietary background (interaction *P* = 0.861). The offspring growth curve is shown in Fig 3B.

Our previous studies have shown that despite no obvious effects on offspring weight, maternal obesity in Swiss mice resulted in significant detrimental effects on mitochondrial ultrastructure in the muscles [48] and fully grown oocytes [25] of the offspring. Here we further investigate the impact on mitochondrial morphology in the preantral stages of folliculogenesis.

### Mitochondrial ultrastructure within oocytes of primordial, primary and secondary follicles in C-born C-fed pups (C»C)

Since studies involving mitochondrial morphology in young developing follicles are lacking, it was necessary to first analyze and compare oocyte mitochondrial morphology during early folliculogenesis in primordial, primary and secondary follicles of adult control offspring (C»C), before dietary effects were investigated. The mitochondrial classifications detected in C»C, and used throughout the study are described in Table 1.

**Table 1:** Different mitochondrial classifications recorded in oocytes within primordial, primary, and secondary follicles in ovaries of C»C pups.

Mitochondrial class	Description of the mitochondria
**Spherical**:	
Full Cristae (FCr)	Abundant distinct cristae and a translucent/light matrix
Sparse Cristae (SCr)	A few distinct cristae and a light matrix
No Cristae (NCr)	Mitochondria without cristae and a light matrix
Dense (D)	An electron dense matrix without distinct cristae
Dense-vacuolated (DV)	An electron dense matrix with a vacuole and no distinct cristae
**Non-spherical**:	Pear, Elongated and Dumbbell-shaped mitochondria
**Abnormal**	Mitochondria showing loose inner membranes, broken or protruded outer membranes, rose-petal shaped mitochondria, enlarged mitochondria, and mitochondria having electron dense foci

**Fig 4:**
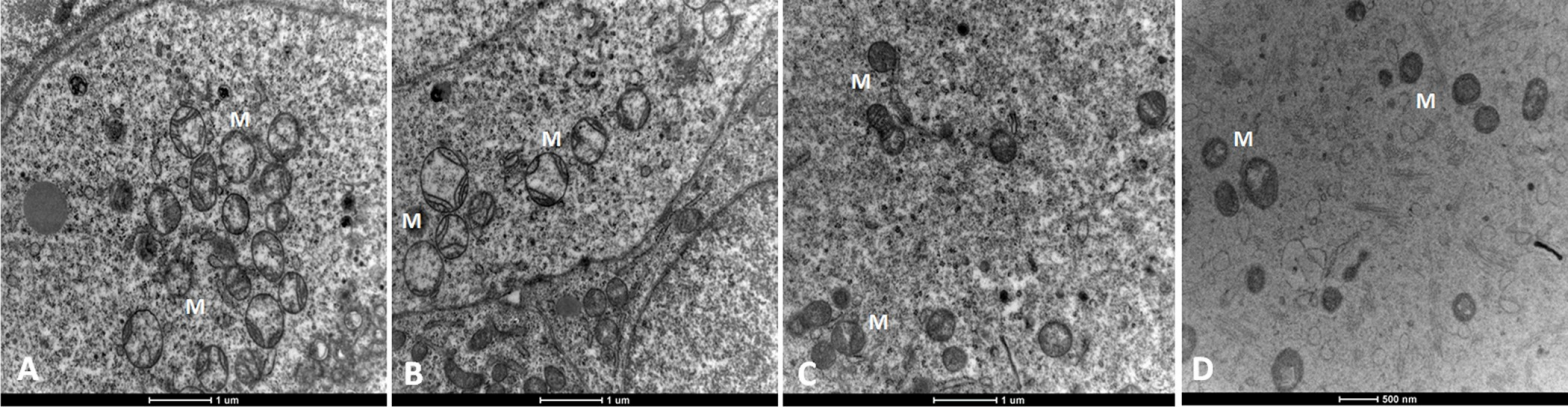
Oocyte mitochondria (M) in a primordial (**A**), primary (**B**) and secondary (**C**) follicle. **D.** Oocyte mitochondria in a mature ovulated oocyte.

Unlike the mitochondrial ultrastructure in mature oocytes (after ovulation) reported in other studies and displayed here in Fig 4, the majority of the oocyte mitochondria within primordial follicles were characterized by low electron density and a translucent or light matrix, with distinct but sparse cristae present. After follicle activation, a proportion of oocyte mitochondria became electron dense, without distinct cristae (Fig 4).

**Fig 5:**
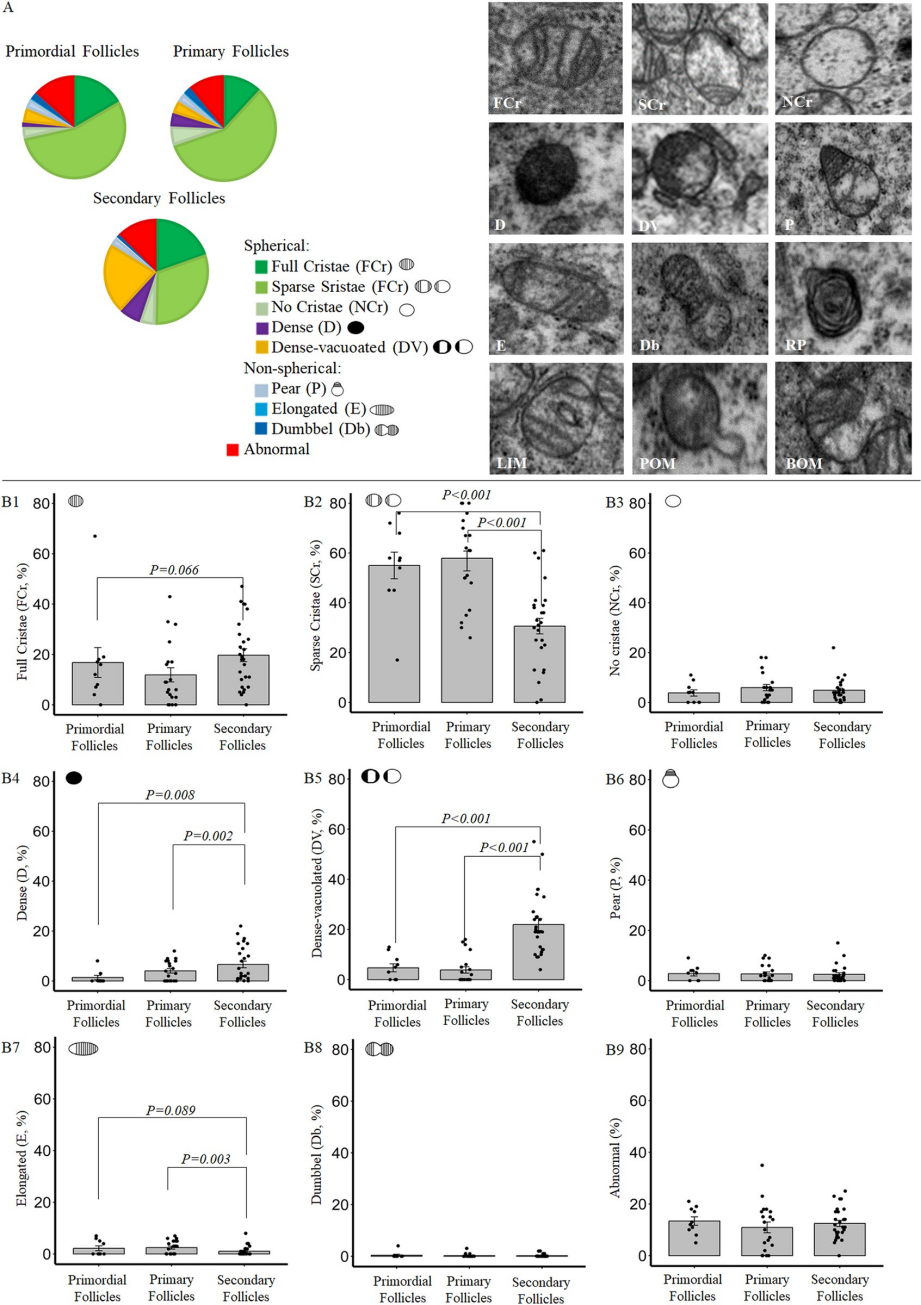
**A**. Description of the oocyte mitochondrial ultrastructure (Pie-charts) in primordial, primary and secondary follicles, and corresponding TEM. Pictures of different mitochondrial subcategories (FCr = Full cristae, SCr = Sparse cristae, NCr = No Cristae, D = Dense, DV = Dense-vacuolated, P = pear-shaped, E = elongated, Db = dumbbell-shaped, RP = rose-petal shaped, LIM = loose inner membrane, BOM = broken outer membrane, POM = protruded outer membrane) in control (C»C) ovaries. **B**. Changes in the proportion of each morphological class between different stages of early follicle development in control (C»C) ovaries. Data are presented as proportion percentages ± S.E. and are derived from 10 primordial, 20 primary and 27 secondary follicles of 6 C»C mice. *P*-values of the significant differences and tendencies are displayed on the graphs.

The majority of the mitochondria in the oocytes of the **primordial ovarian follicles** of adult control (C»C) mice were spherical with light electron density containing full (FCr, 16.8 ± 5.9%), sparce (SCr, 55 ± 5.3%) or no cristea (NCr, 3.8 ± 1.3%) (Fig 5). Electron dense mitochondria were not abundant (1.4 ± 0.8% D and 4.7 ± 1.6% DV). Only 5.4 ± 1.1% of the mitochondria were non-spherical, and 13.4 ± 1.6% of the mitochondria showed ultrastructural abnormalities (listed in Table 1).

Oocytes of the primordial follicles remain arrested at the diplotene stage of the first meiotic division from shortly before birth and until the follicle is activated. They form a limited stock of dormant primordial follicle pool which is crucial for fertility and reproductive longevity after puberty [49–51]. Odor and Blandau [52] studied oocyte mitochondrial morphology in murine primordial follicles from prenatal day 18 until postnatal day 3. In this study, the oocyte mitochondrial morphology in the dormant primordial follicle pool was described to be translucent, with sparse cristae [52]. This specific mitochondrial morphology is also similar to the predominant feature that we observed here in oocytes of primordial follicles at adulthood (age 10 weeks) and suggests that the mitochondrial morphology in these follicles does not change with advancing age.

Cinco [53] suggested, while studying *ex-vivo* neonatal and prepuberal murine ovaries, that oocytes within primordial follicles exhibit elevated free/bound auto-fluorescent NADH ratios, often associated with a reduced cellular respiration [54]. Therefore, the presence of large proportions of mitochondria with low electron density and only few cristae, as detected in our study, may imply an immature oxidative phosphorylation capacity. Oocytes at this stage of development are arrested and do not undergo cytoplasmic or nuclear changes. Thus, these oocytes are not in need of high-energy provision. Throughout this dormant stage they even uphold an adapted mitochondrial metabolism, characterized by low ROS levels through the inhibition of complex I, hereby ensuring minimal cellular stress levels and long-lasting viability [55].

In **primary follicles** of C»C, we found that the proportions of different classes of oocyte mitochondrial morphology were very similar to those at the primordial stage; with spherical, low electron dense mitochondria with full (FCr, 11.9 ± 2.8%) or sparce cristae (SCr, 57.9 ± 4.4%) being most abundant (Fig 5).

In contrast, oocytes of the **secondary follicles** exhibited drastic changes in mitochondrial morphology compared to earlier stages. In these oocytes, SCr mitochondria were still the most predominant (30.6 ± 3.1%), but their presence was reduced by over 25%, along with an increase in the proportions of high electron dense mitochondria (around 18% increase in total, 6.6 ± 1.3% D and 22 ± 2.4% DV) compared to oocytes of earlier stages (primordial and primary) (Fig 5).

Follicle activation initiates oocyte growth and volume expansion [28], accompanied by increased metabolic activity, energy production and concomitant mitochondrial biogenesis [27]. During this process, the oocyte’s metabolism gradually shifts from a mainly glycolytic metabolism to a combination of glycolytic and aerobic metabolism, involving increased oxidative phosphorylation [53] and substantial changes in mitochondrial dynamics [27]. As follicles mature to the secondary stage, the oocyte starts to rely on highly glycolytic cumulus cells as a source of pyruvate, while continuing to be active in oxidative phosphorylation [53,56]. Since oxidative phosphorylation involves electrons being transported over the inner mitochondrial membrane to produce ATP [57], we would expect an increase in the amount of cristae, and thus an increase in the proportion of FCr mitochondria. However, only dense mitochondria (D and DV) were significantly increased in secondary follicle oocytes, as in this stage the subtle increase in the proportion of D and DV mitochondria seen in the primary follicles became apparent. Interestingly, these dense mitochondria look similar to the majority of the mitochondria in mature oocytes [20,36,58], where the matrix is also electron dense. The mitochondrial matrix contains essential components such as DNA, ribosomes, enzymes and co-factors needed for the citric acid cycle and oxidative phosphorylation [59,60]. In mature oocytes, while all mitochondria exhibit electron dense matrix and no or very few distinct cristae, these oocytes exhibit a high mitochondrial membrane potential, and detectable O_2_-linked ATP production as shown using Seahorse analysis [61,62]. Thus, the increase in mitochondrial matrix density detected in secondary follicle oocytes may be linked with an increased mitochondrial activity [21]. However, high electron density of the mitochondrial matrix may also obscure the distinction of cristae.

A small but significant 1% reduction in the proportion of elongated mitochondria was seen in the oocytes as they transit from the primordial to the secondary follicle stage (1.1 ± 0.4% in oocytes of secondary follicles vs 2.8 ± 0.9% and 2.5 ± 0.6% in oocytes of primordial and primary follicles respectively). However, it is only after the blastocyst stage (5–8 days after fertilisation) that the mitochondria of the embryo will gradually transform from immature spherical to elongated mitochondria with clear cristae, as found in somatic cells [63]. In mature oocytes, elongated mitochondria are described as abnormal and indicative of a disturbed fusion/fission machinery [64], which may imply alterations in energy metabolism [36,65]. Nevertheless, it remains questionable if a decrease of only 1%, as seen in our results, can be linked with changes in mitochondrial biogenesis or bioenergetic functions.

### The effect of the maternal and offspring diets on oocyte mitochondrial morphology in offspring preantral ovarian follicles

Following the initial examination of oocyte mitochondrial morphology in preantral follicles of healthy adult mice (C»C), we proceeded to explore the impact of maternal and offspring OB diets and their interaction.

### The effect of the maternal diet on the oocyte mitochondrial morphology of the primordial follicles

**Fig 6:**
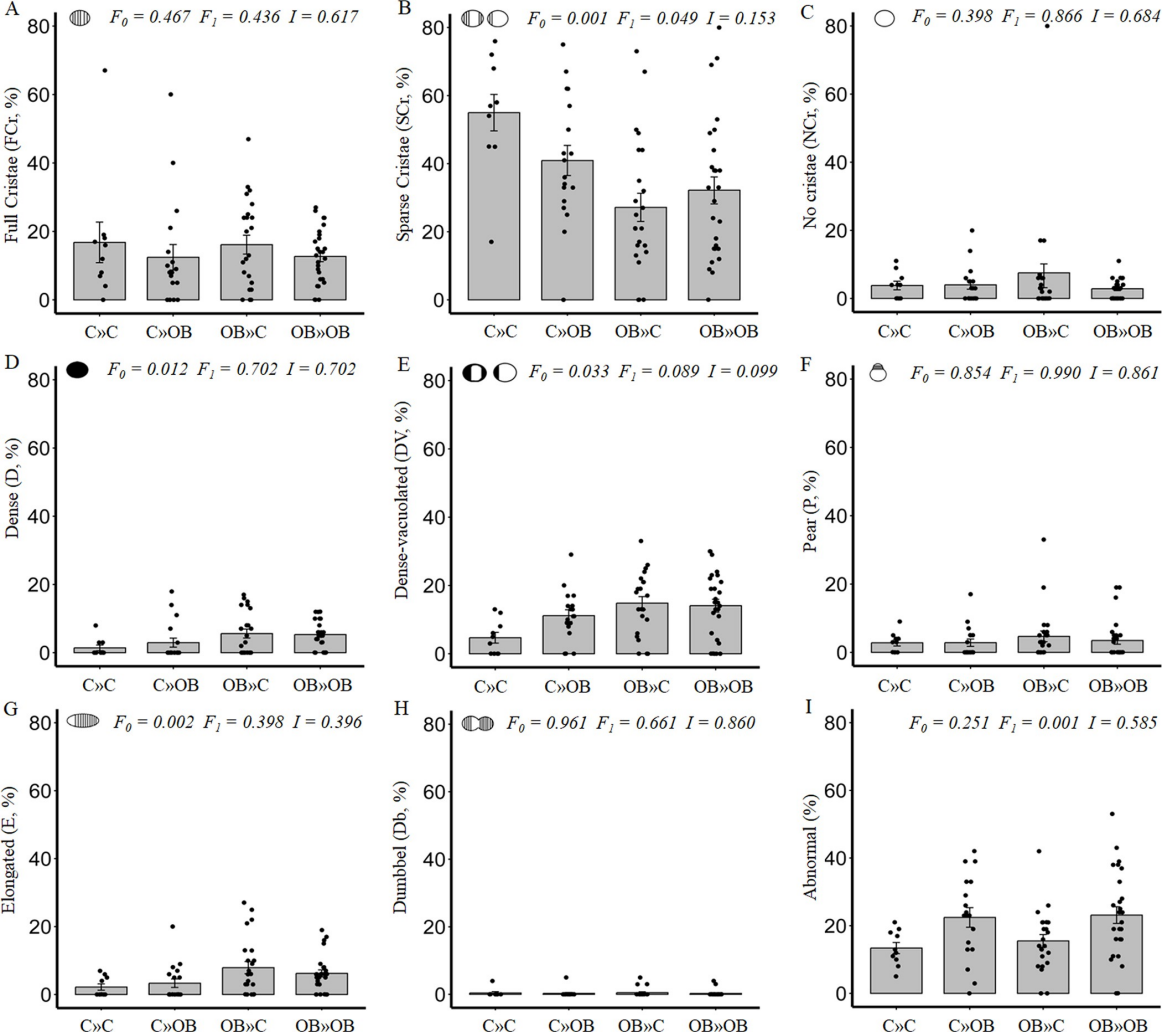
The effect of maternal and offspring OB diets and their interaction on mitochondrial ultrastructure in oocytes of primordial follicles within ovaries of offspring fed a C or an OB diet and born to mothers that were either fed a C or OB diet, in a 2-by-2 factorial design. Data are presented as percentage ± S.E. and are derived from primordial follicles of 6 mice per treatment group (C»C n = 10, C»OB n = 18, OB»C n = 23, OB»OB n = 27 follicles). *P*-values of the main effects are stated (F_0_ = maternal diet effect, F_1_ = offspring diet effect, I = effect of the interaction).

In primordial follicles, maternal OB diet resulted in a significant reduction (by about 18%) of the proportion of mitochondria with low electron-density (particularly with SCr) (C»C: 55.0 ± 5.3%; C»OB: 40.9 ± 4.4%; OB»C: 27.2 ± 4.1%, OB»OB: 32.2 ± 4.0%), associated with a corresponding increase (by up to 10%) in dense mitochondria with vacuoles (DV, C»C: 4.7 ± 1.6%; C»OB: 11.2 ± 1.7%; OB»C 14.8 ± 1.9%; OB»OB 14.1 ± 1.9%) and without vacuoles (D, C»C: 1.4 ± 0.8%; C»OB: 2.9 ± 1.3%; OB»C 5.6 ± 1.3%; OB»OB 5.3± 0.8%, Fig 6).

Such maternal diet induced change in mitochondrial electron density in the primordial follicle oocytes is similar to that observed after follicle activation at the secondary stage in control (C»C) oocytes, and was previously associated with an increased cellular respiration. This may imply an earlier onset of oxidative phosphorylation, or activation of the mitochondria in the primordial follicles of the offspring born to obese mothers.

The maternal OB diet also slightly increased the proportion of elongated mitochondria with 4–5% (C»C: 2.2 ± 0.9%; C»OB: 3.3 ± 1.3%; OB»C: 7.9 ± 1.8%; OB»OB: 6.2 ± 1.0%). Mitochondrial elongation is associated with changes in energy efficiency and may even indicate alterations in mitochondrial dynamics and replication, as described by others [36,65,66].

Interestingly, only the presence of cristae, differences in mitochondrial density or shape were affected by a maternal OB diet, and may imply rather functional adaptations instead of dysfunction [47]. The proportions of mitochondrial abnormalities, directly associated with mitochondrial dysfunction [20] remained unaffected by a maternal OB diet.

It is suggested that mitochondrial dysfunction in oocytes of offspring born to obese mothers may originate from the maternal aberrant oocyte mitochondria that are transferred to the embryo at conception, due to the inability of oocytes to activate mitophagy [36,67]. However, these studies were performed in inbred C57BL/6 mice, while it is known that C57BL/6 mice already show an increased rate of inborn oocyte mitochondrial ultrastructural abnormalities (compared to Swiss mice of the same age, fed the same control diet), which may affect the mitochondrial transmission to the next generations [20]. On top, several mechanisms preclude such transfer of mitochondrial damage to the offspring, e.g. the bottleneck phenomenon and mitochondrial self-repair [68,69]. Multiple rounds of mitochondrial segregation and replication during the formation of the primordial follicle pool even increase the chance of inducing *de-novo* mutations [70,71], making this germline transmission of mitochondrial dysfunction rather unlikely. Presumably, the maternal OB diet induced alterations that we detected in the oocytes of the offspring’s primordial follicle pool, may occur due to the direct effects of the maternal OB diet on the dividing primordial germ cells during pregnancy, drastically impacting the early formation of the primordial follicle pool, or even affect the offspring’s oocyte mitochondria post-gestational during lactation. Currently, we are trying to make similar assessments to investigate if these primordial follicle pool oocytes are already affected at birth.

#### The effect of the offspring diet on the oocyte mitochondrial morphology of the primordial follicles

In contrast with maternal diet effects, offspring OB diet significantly increased the proportion of abnormal mitochondria in the primordial follicle oocytes by around 10% (C»C: 13.4 ± 1.6%; C»OB: 22.4 ± 2.9%; OB»C: 15.5 ± 1.9%; OB»OB: 23.1 ± 2.5%). Offspring diet also had a significant effect on the proportions of SCr mitochondria (C»C: 55.0 ± 5.3%; C»OB: 40.9 ± 4.4%; OB»C: 27.2 ± 4.3; OB»OB: 32.2 ± 4.0%) and a tendency to increase the proportion of dense mitochondria. However these effects were only obvious in offspring born to mothers fed a control diet, and may be masked by maternal obesity. Nevertheless, the interaction between offspring and maternal diet effects on SCr and D mitochondria was never significant (Fig 6).

The mitochondrial abnormalities detected in our study involved mitochondria with protruded or ruptured outer membrane, which may suggest mitochondrial bursting. Mitochondrial bursting indicates exacerbated levels of oxidative stress, ultimately leading to complete mitochondrial dysfunction [72,73]. Also, loose inner membrane structures inside an enlarged intercristal space were detected, previously described as an abnormal mitochondrial feature in mature oocytes [20,25]. Mitochondria with electron dense foci and rose-petal shaped mitochondria were also classified here as abnormal. Others associated the presence of electron dense foci with altered glucose and oxygen metabolism [74], and rose-petal shaped mitochondria are linked with affected ATP-synthase [75,76] which is a major component of the fifth mitochondrial complex, needed to produce energy [77]. Our results thus indicate that offspring OB diet is detrimental for oocyte quality in the primordial follicles. Most direct effects of an OB diet on fully grown (matured or ovulated) oocytes are attributed to biochemical changes in the composition of the follicular fluid, such as increased lipid concentrations and pro-inflammatory cytokines [17–19]. With our results, we show for the first time that these effects may already be induced even before the formation of the antrum.

#### Effects of maternal and offspring diets on mitochondria in primary and secondary follicles

**Fig 7:**
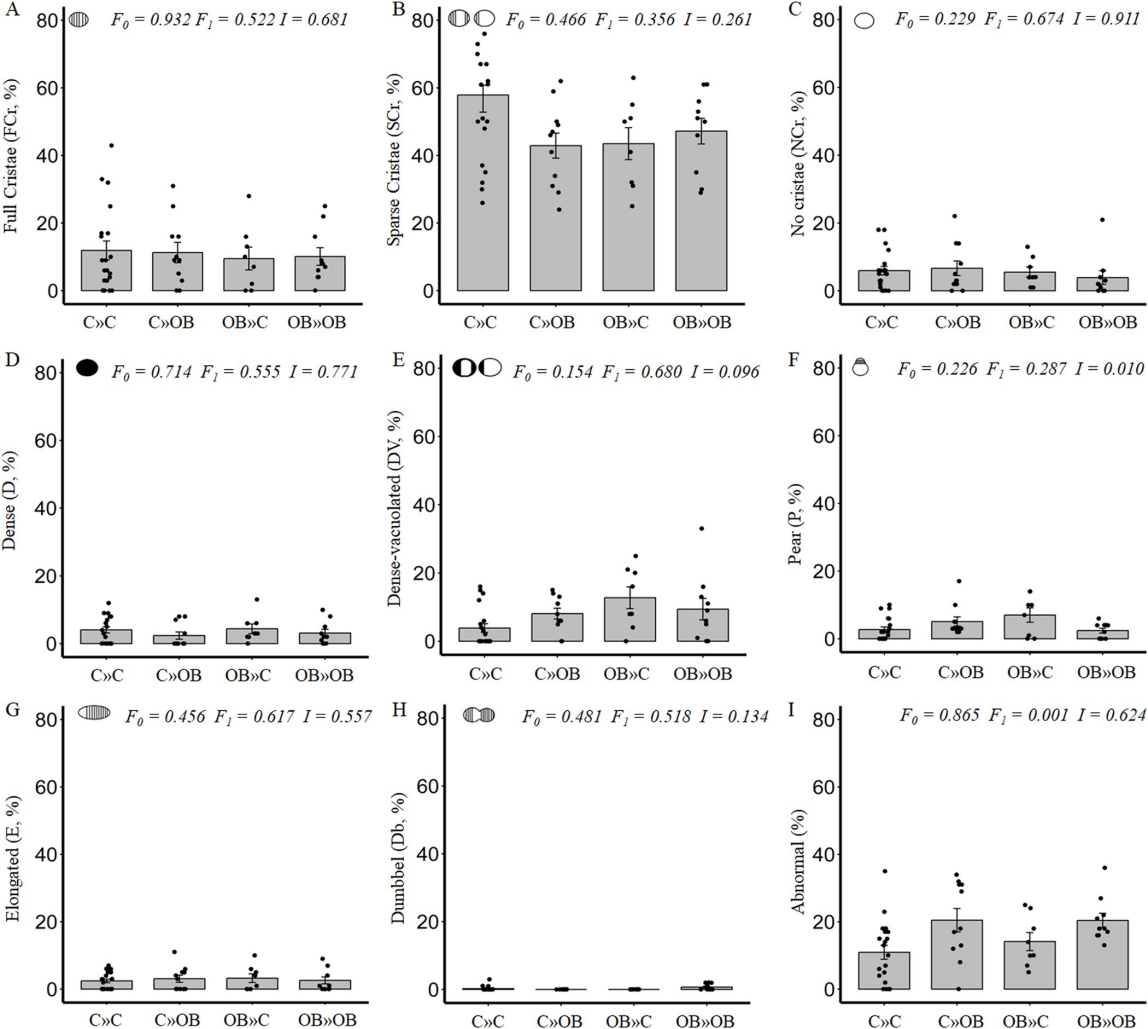
The effect of maternal and offspring OB diets and their interaction on mitochondrial ultrastructure in oocytes of primary follicles within ovaries of offspring fed a C or an OB diet and born to mothers that were either fed a C or OB diet, in a 2-by-2 factorial design. Data are presented as percentage ± S.E. and are derived from primary follicles of 6 mice per treatment group (C»C n = 20, C»OB n = 11, OB»C n = 8, OB»OB n = 10 follicles). *P*-values of the main effects are stated (F_0_ = maternal diet effect, F_1_ = offspring diet effect, I = effect of the interaction).

**Fig 8:**
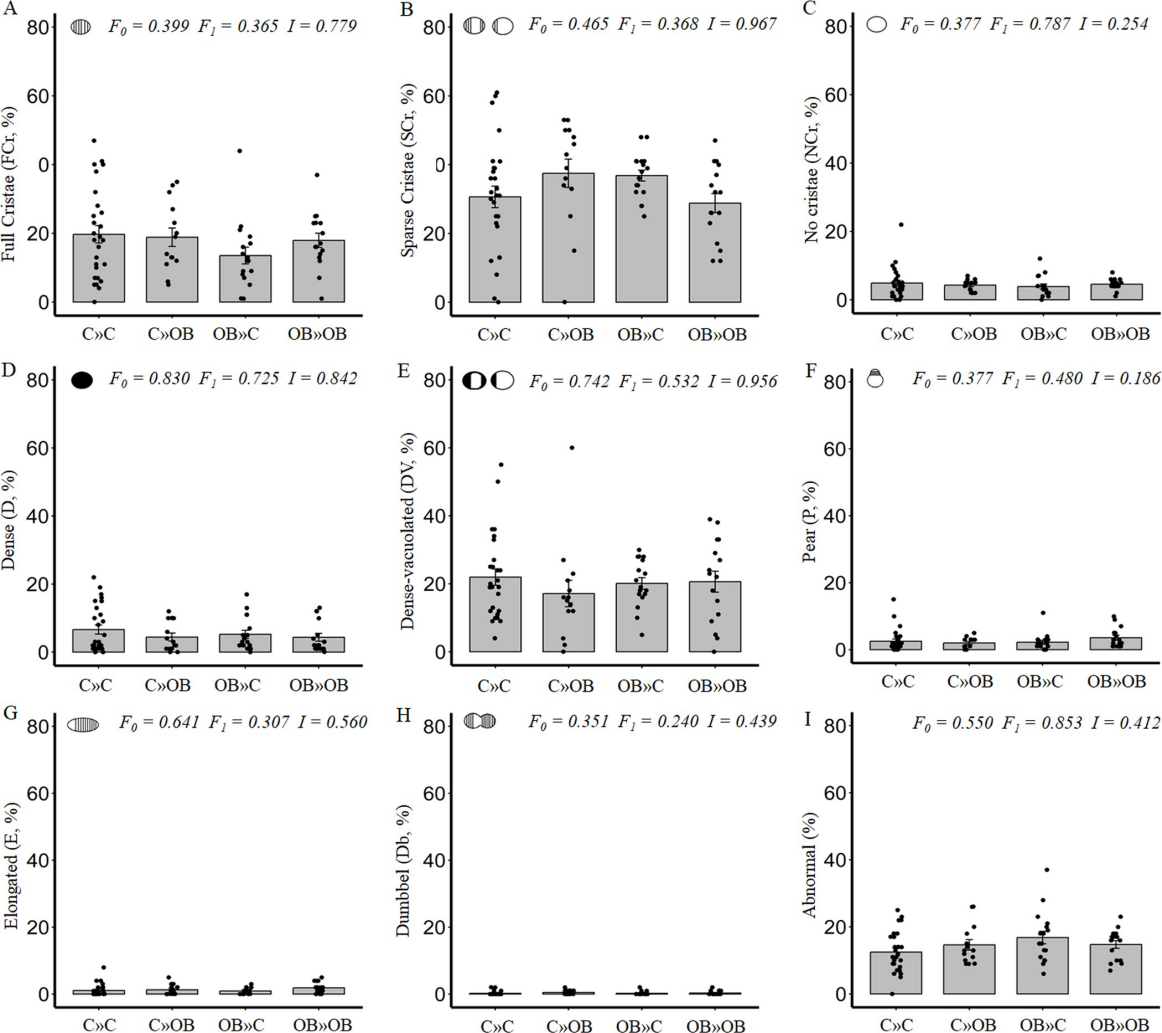
The effect of maternal and offspring OB diets and their interaction on mitochondrial ultrastructure in oocytes of secondary follicles within ovaries of offspring fed a control (C) or an obesogenic diet (OB) and born to mothers that were either fed a C or OB diet, in a 2-by-2 factorial design. Data are presented as percentage ± S.E. and are derived from secondary follicles of 6 mice per treatment group (C»C n = 27, C»OB n = 14, OB»C n = 17, OB»OB n = 16 follicles). *P*-values of the main effects are stated (F_0_ = maternal diet effect, F_1_ = offspring diet effect, I = effect of the interaction).

We could not detect any effects of the maternal OB diet on oocyte mitochondrial ultrastructure in the primary and secondary follicles (Figs 7 and 8 respectively). This could be due to changes in the oocyte mitochondrial morphology that already occur when the primordial follicles are activated (as described above), which may mask the detection of maternal diet effects. However, while follicle atresia was not assessed in this study, the absence of dietary effects in these late stages of preantral follicular development may also imply that only the follicles with the best oocyte mitochondria are able to reach the secondary stage. Nevertheless, further research is required to understand these notions at a functional level.

Similarly, we could not detect any effect of the offspring OB diet on mitochondrial density or shape in the primary and secondary oocytes (Figs 7 and 8 respectively). However, the effects on the proportions of abnormal mitochondria in primary oocytes were almost identical to those observed in the primordial follicles (C»C: 11.0 ± 2.1%; C»OB: 20.5 ± 3.5%; OB»C: 14.1 ± 2.7%; OB»OB: 20.4 ± 2.1%), and indicative of mitochondrial damage.

A parallel study done at our lab, using the same mouse model and experimental design but focusing on the dietary impact on fully grown mature oocytes (collected after ovulation), clearly showed that the direct effect of the offspring’s OB diet on mitochondrial ultrastructure in these mature ovulated oocytes was influenced by the maternal metabolic background [25]. Therefore, the dietary effects on the mitochondrial ultrastructure detected here in the offspring’s primordial follicles may have long-term repercussions throughout subsequent folliculogenesis.

## Conclusions

In conclusion, this study characterized and described the oocyte mitochondrial morphology in primordial and preantral follicles. We showed that the matrix density of primordial follicle oocyte mitochondria increases after follicle activation, thereby becoming similar to the mitochondrial morphology of the well described mature (ovulated) oocytes. On top, the effect of both maternal and offspring OB diets and their interaction on oocyte mitochondria in primordial and early activated follicles was investigated using a pathophysiological relevant outbred mouse model in a 2-by-2 factorial design, thereby mimicking the human situation of female familial obesity. We are the first to show that the oocyte mitochondria of the dormant primordial follicle pool are already affected by both maternal and offspring OB diets, which may impact the quality of the oocyte during subsequent stages of folliculogenesis. While our study remains descriptive, focusing only on the mitochondrial morphology without functional analysis, it was clear that the maternal OB diet increased the mitochondrial density already in the primordial follicles, suggesting an earlier increase in bioenergetic capacity. Interestingly, maternal obesity did not induce abberant oocyte mitochondrial ultrastructure (abnormalities and defects) in preantral follicles of their offspring, and did not influence the direct effect of an offspring OB diet. In contrast, only offspring OB diet increased the oocyte mitochondrial abnormalities in the primordial follicles, indicative for mitochondrial damage. While further functional investigations are still required, the unique and novel insights provided here at these very early stages strongly suggests that preantral folliculogenesis is a crucial window of sensitivity of the oocyte for the effects of maternal and offspring OB diets, which may require attention when developing or optimizing reproductive managing protocols for obese patients.

## Supporting information

S1 Fig TEM overview of apoptotic follicles. A. overview of an apoptotic follicle; B. fragmentation of the oocyte within the apoptotic follicle; C. close-up image of mitochondria in the oocyte within an apoptotic follicle (O = oocyte; M = mitochondria within the oocyte cytoplasm; ZP = zona pellucida; GC = granulosa cells, F = oocyte fragmentation, CG = cortical granule, LD = lipid droplet). Whereas the mitochondria in oocytes of non-atretic follicles were more or less equally dispersed throughout the cytoplasm, the mitochondria in oocytes of apoptotic follicles were not. Only in these follicles, signs of mitochondrial clustering were detected with cortical granules present in the peri-cortical area of the oocyte. Lipid droplets were seen in the oocyte and in the surrounding granulosa cells. The oocyte itself was irregularly shaped, showing signs of fragmentation.
(TIF)

S1 File (DOCX)
