# Supplementary figures and images for: The impact of offspring and maternal obesogenic diets on adult offspring oocyte mitochondrial morphology in primordial and preantral follicles
Source: PLoS One. 2024 Jun 27;19(6):e0305912. doi: 10.1371/journal.pone.0305912 (PMC11210809; doi:10.1371/journal.pone.0305912)

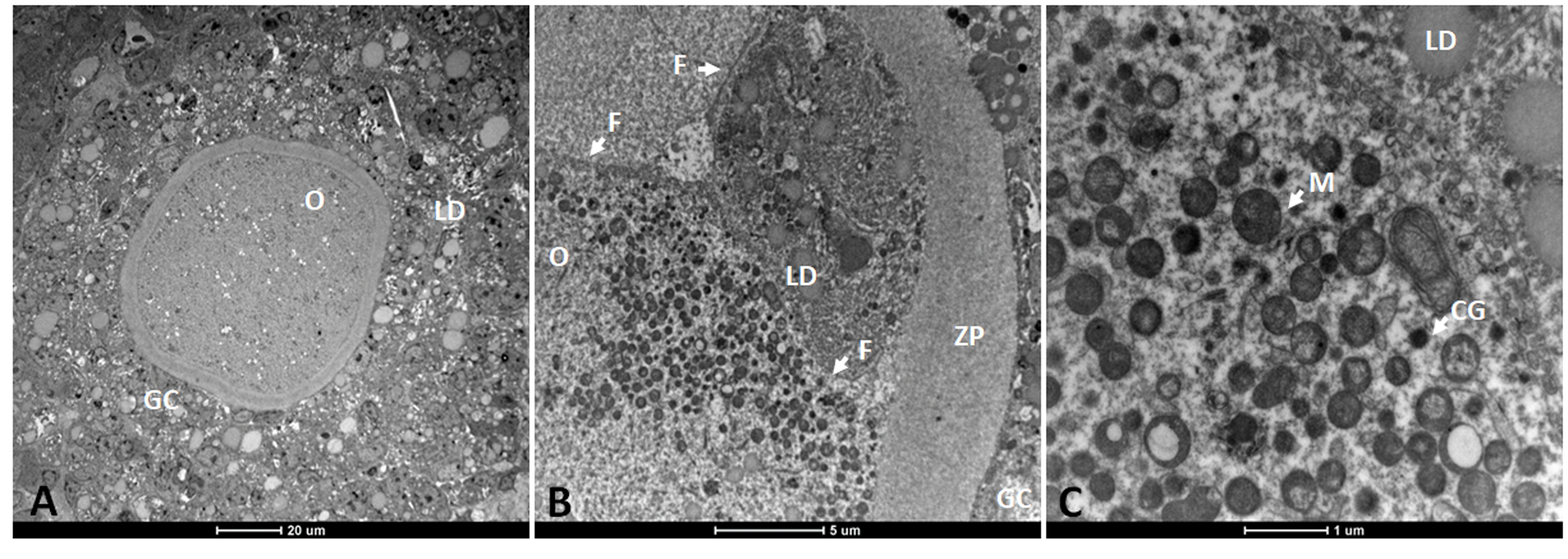

Supplement: S1 Fig — A. overview of an apoptotic follicle; B. fragmentation of the oocyte within the apoptotic follicle; C. close-up image of mitochondria in the oocyte within an apoptotic follicle (O = oocyte; M = mitochondria within the oocyte cytoplasm; ZP = zona pellucida; GC = granulosa cells, F = oocyte fragmentation, CG = cortical granule, LD = lipid droplet). Whereas the mitochondria in oocytes of non-atretic follicles were more or less equally dispersed throughout the cytoplasm, the mitochondria in oocytes of apoptotic follicles were not. Only in these follicles, signs of mitochondrial clustering were detected with cortical granules present in the peri-cortical area of the oocyte. Lipid droplets were seen in the oocyte and in the surrounding granulosa cells. The oocyte itself was irregularly shaped, showing signs of fragmentation. (TIF) [file pone.0305912.s001.tif]
