# Supplementary material for: The impact of offspring and maternal obesogenic diets on adult offspring oocyte mitochondrial morphology in primordial and preantral follicles
Source: PLoS One. 2024 Jun 27;19(6):e0305912. doi: 10.1371/journal.pone.0305912 (PMC11210809; doi:10.1371/journal.pone.0305912)
Supplement: S1 File — (DOCX) [file pone.0305912.s002.docx]

**S1 Table 1.** Amount of mitochondria studied in primordial follicles in all treatment groups.

|  | Mean number of oocyte mitochondria per follicle ± S.E.M | Maximum number of mitochondria per follicle | Minimum number of mitochondria per follicle |
| --- | --- | --- | --- |
| C»C | 24.1 ± 3.2 | 38 | 6 |
| C»OB | 20.4 ± 2.5 | 39 | 3 |
| OB»C | 24.3 ± 2.8 | 49 | 1 |
| OB»OB | 23.8 ±1.8 | 49 | 11 |

Data are derived from all primordial follicles of 6 mice per treatment group (C»C n = 10, C»OB n = 18, OB»C n = 23, OB»OB n = 27 follicles).

**S1 Table 2.** Amount of mitochondria studied in primary follicles in all treatment groups.

|  | Mean number of oocyte mitochondria per follicle ± S.E.M | Maximum number of mitochondria per follicle | Minimum number of mitochondria per follicle |
| --- | --- | --- | --- |
| C»C | 40.2 ± 4.9 | 91 | 11 |
| C»OB | 36.5 ± 3.6 | 63 | 19 |
| OB»C | 49.9 ± 11.4 | 97 | 8 |
| OB»OB | 61.3 ± 8.4 | 99 | 15 |

Data are derived from all primary follicles of 6 mice per treatment group (C»C n = 20, C»OB n = 11, OB»C n = 8, OB»OB n = 10 follicles).

**S1 Table 3.** Amount of mitochondria studied in secondary follicles in all treatment groups.

|  | Mean number of oocyte mitochondria per follicle ± S.E.M | Maximum number of mitochondria per follicle | Minimum number of mitochondria per follicle |
| --- | --- | --- | --- |
| C»C | 91.2 ± 6.7 | 157 | 31 |
| C»OB | 125.6 ± 9.6 | 202 | 60 |
| OB»C | 117.2 ± 11.3 | 175 | 26 |
| OB»OB | 136.7 ± 9.6 | 199 | 65 |

Data are derived from all secondary follicles of 6 mice per treatment group (C»C n = 27, C»OB n = 14, OB»C n = 17, OB»OB n = 16 follicles).

**S2 Table 1:** Different mitochondrial phenotypes of primordial follicles per treatment group primordial.

|  | FCr | SCR | NCr | D | DV | P | E | DB | abnormal |
| --- | --- | --- | --- | --- | --- | --- | --- | --- | --- |
| C»C | 0.168±0.059 | 0.550±0.053 | 0.038±0.013 | 0.014±0.008 | 0.047±0.016 | 0.028±0.009 | 0.022±0.009 | 0.004±0.004 | 0.134±0.016 |
| C»OB | 0.124±0.037 | 0.409±  0.044 | 0.040±0.013 | 0.029±0.013 | 0.112±0.017 | 0.028±0.011 | 0.033±0.013 | 0.003±0.003 | 0.224±0.029 |
| OB»C | 0.161±0.028 | 0.272±0.041 | 0.075±0.043 | 0.056±0.013 | 0.148±0.019 | 0.047±0.016 | 0.079±0.018 | 0.05±0.003 | 0.155±0.019 |
| OB»OB | 0.127±0.016 | 0.322±0.040 | 0.029±0.006 | 0.053±0.008 | 0.141±0.019 | 0.035±0.011 | 0.062±0.010 | 0.003±0.002 | 0.231±0.025 |

The groups are named as MaternalDiet»OffspringDiet. Mitochondria showing loose inner membranes, broken or protruded outer membranes, rose-petal shaped mitochondria, enlarged mitochondria (>1µm), and electron dense mitochondria as described by Marei [20], are classified as abnormal. Data are presented as proportion±S.E. and are derived from all primordial follicles of 6 mice per treatment group (C»C n = 10, C»OB n = 18, OB»C n = 23, OB»OB n = 27 follicles).

**S2 Table 2**: Different mitochondrial phenotypes of primary follicles per treatment group.

|  | FCr | SCr | NCr | D | DV | P | E | DB | abnormal |
| --- | --- | --- | --- | --- | --- | --- | --- | --- | --- |
| C»C | 0.119±0.028 | 0.579±0.044 | 0.060±0.013 | 0.041±0.009 | 0.039±0.013 | 0.028±0.009 | 0.025±0.006 | 0.003±0.002 | 0.110±0.021 |
| C»OB | 0.113±0.030 | 0.429±0.037 | 0.066±0.021 | 0.024±0.011 | 0.081±0.016 | 0.051±0.014 | 0.031±0.011 | 0.000±0.000 | 0.205±0.035 |
| OB»C | 0.095±0.034 | 0.435±0.047 | 0.055±0.015 | 0.044±0.014 | 0.128±0.032 | 0.070±0.021 | 0.033±0.013 | 0.000±0.000 | 0.141±0.027 |
| OB»OB | 0.101±0.026 | 0.472±0.038 | 0.039±0.020 | 0.031±0.011 | 0.094±0.032 | 0.042±0.007 | 0.026±0.010 | 0.007±0.003 | 0.204±0.021 |

## The groups are named as MaternalDiet»OffspringDiet. Mitochondria showing loose inner membranes, broken or protruded outer membranes, rose-petal shaped mitochondria, enlarged mitochondria (>1µm), and electron dense mitochondria as described by Marei [20], are classified as abnormal. Data are presented as proportion±S.E. and are derived from all primary follicles of 6 mice per treatment group (C»C n = 20, C»OB n = 11, OB»C n = 8, OB»OB n = 10 follicles).

**S2 Table 3**: Different mitochondrial phenotypes of secondary follicles per treatment group.

|  | FCr | SCr | NCr | D | DV | P | E | DB | abnormal |
| --- | --- | --- | --- | --- | --- | --- | --- | --- | --- |
| C»C | 0.197±0.025 | 0.306±0.031 | 0.049±0.009 | 0.066±0.013 | 0.220±0.024 | 0.025±0.007 | 0.011±0.004 | 0.002±0.001 | 0.125±0.012 |
| C»OB | 0.189±0.027 | 0.375±0.041 | 0.043±0.004 | 0.044±0.012 | 0.171±0.039 | 0.020±0.004 | 0.013±0.004 | 0.005±0.002 | 0.146±0.016 |
| OB»C | 0.135±0.024 | 0.368±0.016 | 0.039±0.007 | 0.052±0.012 | 0.201±0.017 | 0.022±0.006 | 0.009±0.002 | 0.002±0.001 | 0.168±0.0019 |
| OB»OB | 0.179±0.02 | 0.288±0.028 | 0.046±0.004 | 0.044±0.012 | 0.206±0.031 | 0.036±0.007 | 0.019±0.004 | 0.003±0.002 | 0.148±0.011 |

The groups are named as MaternalDiet»OffspringDiet. Mitochondria showing loose inner membranes, broken or protruded outer membranes, rose-petal shaped mitochondria, enlarged mitochondria (>1µm), and electron dense mitochondria as described by Marei [20], are classified as abnormal. Data are presented as proportion±S.E. and are derived from all secondary follicles of 6 mice per treatment group (C»C n = 27, C»OB n = 14, OB»C n = 17, OB»OB n = 16 follicles).
